# The Role of *β3*-Adrenergic Receptors in Cold-Induced Beige Adipocyte Production in Pigs

**DOI:** 10.3390/cells13080709

**Published:** 2024-04-19

**Authors:** Shuo Yang, Hong Ma, Liang Wang, Fang Wang, Jiqiao Xia, Dongyu Liu, Linlin Mu, Xiuqin Yang, Di Liu

**Affiliations:** 1 College of Animal Science and Technology, Northeast Agricultural University, Harbin 150030, China; 2 Institute of Animal Husbandry, Heilongjiang Academy of Agricultural Sciences, Harbin 150086, China; 3 College of Animal Science and Technology, Hebei Normal University of Science & Technology, Qinhuangdao 066004, China; 4 College of Animal Science and Veterinary Medicine, Heilongjiang Bayi Agricultural University, Daqing 163319, China; 5 Institute of Forage and Grassland Sciences, Heilongjiang Academy of Agricultural Sciences, Harbin 150086, China

**Keywords:** cold stress, piglets, *ADRB3*, beige adipocytes, thermogenesis

## Abstract

After exposure to cold stress, animals enhance the production of beige adipocytes and expedite thermogenesis, leading to improved metabolic health. Although brown adipose tissue in rodents is primarily induced by *β3*-adrenergic receptor (*ADRB3*) stimulation, the activation of major *β*-adrenergic receptors (*ADRBs*) in pigs has been a topic of debate. To address this, we developed overexpression vectors for *ADRB1*, *ADRB2*, and *ADRB3* and silenced the expression of these receptors to observe their effects on the adipogenic differentiation stages of porcine preadipocytes. Our investigation revealed that cold stress triggers the transformation of subcutaneous white adipose tissue to beige adipose tissue in pigs by modulating adrenergic receptor levels. Meanwhile, we found that *ADRB3* promotes the transformation of white adipocytes into beige adipocytes. Notably, *ADRB3* enhances the expression of beige adipose tissue marker genes, consequently influencing cellular respiration and metabolism by regulating lipolysis and mitochondrial expression. Therefore, *ADRB3* may serve as a pivotal gene in animal husbandry and contribute to the improvement of cold intolerance in piglets.

## 1. Introduction

Different types of adipose tissue have distinct functions: white adipose tissue (WAT) stores energy, whereas brown adipose tissue (BAT) contains a high number of activated uncoupling protein 1 (*UCP1*) mitochondria that produce heat [1,2]. Subcutaneous white adipose tissue (sWAT) can transition to a beige phenotype, promoting metabolic heat production, in a process known as “browning” [3,4]. Cold exposure is a potent trigger for the recruitment of beige adipocytes [5]. Cold stimulation activates BAT by increasing sympathetic outflow, leading to the uncoupling of the inner mitochondrial membrane, which results in heat production, energy expenditure, and ATP formation, which are crucial for maintaining body temperature in rodents. Additionally, activated BAT in rodent models has been shown to enhance glucose tolerance and insulin sensitivity and to promote weight loss [6].

Throughout evolution, pigs have gradually lost BAT and *UCP1* because their environment and lifestyle render this tissue and protein unnecessary for their survival. Instead, pigs rely more on WAT to store energy. Beige adipocytes are dispersed in WAT in response to various stimuli, such as cold exposure and exercise [7], and are obtained through de novo differentiation of native precursor adipocytes or the reprogramming of mature white adipocytes [8,9,10]. Min pigs, which are grown under cold conditions, have shown resistance to cold; this has been attributed in previous studies to muscle and fat thickness [11,12,13]. However, Zhao et al. observed the presence of beige adipocytes in the adipose tissue of cold-tolerant pig breeds, such as Tibetan and Min pigs [14,15], whereas no beige adipocytes were detected in the adipose tissue of cold-sensitive pigs (Parmesan and Five-Fingered Mountain pigs) after 4 h of cold stimulation. This evidence suggests that cold resistance in Min pigs may be due to the presence of beige adipose tissue.

In rodents, *ADRB3* is the primary adrenergic receptor found in brown adipocytes, and its activation leads to the activation of *UCP1*, thereby increasing oxygen consumption and energy expenditure in BAT cells [16,17]. The physiological role of human *ADRB3* in these processes remains controversial. In contrast, *ADRB1* and *ADRB2* are believed to regulate lipolysis and thermogenesis, respectively, in humans. *ADRB1* has been shown to increase the expression of *UCP1* in human pluripotent stem cells and immortalized brown adipocytes [18], whereas *ADRB2* has recently been implicated as a major regulator of human thermogenesis [19,20]. Studies have demonstrated a close relationship between adrenergic receptor activity and stress response, cardiovascular function, and metabolic regulation in pigs [21,22,23]; however, few studies have been conducted on porcine adipocytes. Despite the significant differences in thermogenesis patterns between pigs and humans due to the lack of *UCP1*, adrenergic receptors may play a crucial role in the beige coloration of porcine white adipocytes.

In rodents, *ADRB3* is the primary adrenergic receptor found in brown adipocytes, and its activation leads to the activation of *UCP1*, thereby increasing oxygen consumption and energy expenditure in BAT cells [16,17]. The physiological role of human *ADRB3* in these processes remains controversial. In contrast, *ADRB1* and *ADRB2* are believed to regulate lipolysis and thermogenesis, respectively, in humans. *ADRB1* has been shown to increase the expression of *UCP1* in human pluripotent stem cells and immortalized brown adipocytes [18], whereas *ADRB2* has recently been implicated as a major regulator of human thermogenesis [19,20]. Studies have demonstrated a close relationship between adrenergic receptor activity and stress response, cardiovascular function, and metabolic regulation in pigs [21,22,23]; however, few studies have been conducted on porcine adipocytes. Despite the significant differences in thermogenesis patterns between pigs and humans due to the lack of *UCP1*, adrenergic receptors may play a crucial role in the beige coloration of porcine white adipocytes.

This study aimed to determine the adrenergic receptors involved in beige coloration in WAT. In vivo and in vitro experiments were conducted to validate the roles of *ADRB1*, *ADRB2*, and *ADRB3* in porcine preadipocytes, revealing that *ADRB3* is essential for the lipolysis and thermogenesis of beige adipocytes under cold stress and highlighting its importance in maintaining beige adipocytes.

## 2. Materials and Methods

### 2.1. Animals and Treatments

The piglets were handled according to the approved guidelines and regulations of the Animal Care Committee of Northeast Agricultural University (Harbin, China). Thirty-day-old piglets were obtained from the experimental farm of the Heilongjiang Academy of Agricultural Sciences. For this study, Min pigs were divided into a control group (temperature = 25 °C, *n* = 5) and a cold exposure group (temperature = 4 °C, *n* = 5). Five male piglets were randomly selected for the experiment, and each treatment group was tested in metabolic cages at 25 °C and 4 °C. During the three days that they were kept in individual metabolism cages with water tanks and feeding troughs for the raising and testing phases, the pigs had unrestricted access to food and water.

### 2.2. Histology

For histological analysis, the adipose tissues were fixed overnight in 4% paraformaldehyde, gradually dehydrated in low to high concentrations of ethanol, and embedded in paraffin. At least 5 slices for each sample were prepared, and a total of 24 slices for each group were screened out and stained with hematoxylin and eosin (H&E). Using a microscope (DP40; OLYMPUS, Tokyo, Japan), images were taken. The adipose tissues were preserved overnight at 4 °C in glutaraldehyde fixative in preparation for transmission electron microscopy (TEM). The samples were dehydrated in increasing ethanol grades, post-fixed in 1% osmium tetroxide, then implanted in brand new epoxy resin. Lead citrate was used to cut and stain extremely thin (60–80 nm) sections before they were examined with a transmission electron microscope (JEM1400, JEOL, Tokyo, Japan).

### 2.3. Quantitative RT-PCR Analysis

The samples were treated using TRIzol Reagent (420810, Ambion, Beijing, China) to extract total RNA. Then, using the PrimeScriptTM RT reagent kit and the gDNA Eraser kit (RR047a, Takara, Beijing, China), total RNA was reverse transcribed into cDNA. To measure mRNA expression, RT-qPCR was carried out using the UltraSYBR One Step RT-qPCR Kit (01170, Cwbio, Beijing, China). Using the 2^−ΔΔCt^ technique, the relative mRNA expression of the target gene was determined and adjusted to that of β-actin. Table S1 provides a summary of all the primer data.

### 2.4. Mitochondrial DNA Copy Number

Total DNA, including genomic DNA and mtDNA, was extracted from adipose tissues and adipocytes using a TIANamp Genomic DNA Kit (DP304, TIANGEN, Beijing, China) following the manufacturer’s instructions. The concentration of DNA was then measured with a biophotometer (B-500, Metash, Shanghai, China). Using an Analytik-Jena qTOWER real-time PCR machine, the mtDNA copy number in relation to the genomic DNA content was quantitatively assessed (Analytik-Jena, Beijing, China).

### 2.5. Isolation, Culture, and Differentiation of Porcine SVF Cells

Five one-month-old, male Min piglets had their inguinal subcutaneous white adipose tissue (IWAT) removed. After the adipose tissue was thoroughly dissected, it was chopped and digested for 90 min at 37 °C in DPBS (SC106, Sevenbio, Beijing, China). The tissue slurry was centrifuged and filtered to pellet SVF cells, which were then suspended in DMEM/F12 media (PM150312, Pricella, Wuhan, China).

Once the SVF cells reached 100% confluence, they were induced in the induction medium to differentiate them. For white adipocyte differentiation, the cells were treated with white adipocyte induction medium (DMEM/F12 medium containing 10% FBS, 0.5 μM insulin) on day 2. For beige adipocyte differentiation, the cells were treated with beige adipocyte induction medium (DMEM/F12 medium containing 10% FBS, 1 nM T3, 0.5 μM insulin, and 2 μM rosiglitazone) on day 2. The induction medium was changed every 2 days.

### 2.6. Overexpression of Porcine ADRB1, ADRB2 and ADRB3

The complete coding regions of porcine *ADRB1* (NM_001123074.1), *ADRB2* (NM_001128436.1), and *ADRB3* (NM_001099927.1) were amplified using porcine cDNA and primers (Supplementary Table S2). Subsequently, the purified PCR products were inserted between the Hind 3 and EcoR I sites of the pcDNA3.1(+) vector (Promega, Madison, WI, USA) to construct pcDNA3.1(+)-*ADRB1*, pcDNA3.1(+)-*ADRB2*, and pcDNA3.1(+)-*ADRB3*. Following construction, the expression of the plasmids was confirmed using DNA sequencing. The SVF cells were then transfected with 1.25 μg of pcDNA3.1(+)-*ADRB1*, pcDNA3.1(+)-*ADRB2*, pcDNA3.1(+)-*ADRB3*, or pcDNA3.1(+). After transfection, all the cells were collected, and RT-qPCR was performed to analyze gene expression.

### 2.7. siRNA Knockdown of ADRB1, ADRB2, and ADRB3

For gene silencing in the SVF cells, commercially available siRNAs specific to *ADRB1*, *ADRB2*, and *ADRB3* (GENERAL BIOL, Wuhan, China) were used. The siRNA sequences used are listed in Table S3. Following the manufacturer’s instructions, Lipofectamine 2000 Transfection Reagent (Thermo Fisher Scientific, Waltham, MA, USA) was used to distribute the siRNAs at a final concentration of 100 nM in the medium. The cells were cultured in DMEM/F12 media (D6421, Sigma, Santa Fe, New Mexico, USA) supplemented with siRNA/Lipofectamine 2000 reagent on the transfection day. After 24 h of transfection, the cells were harvested for gene expression analysis to validate the knockdown of *ADRB1*, *ADRB2*, and *ADRB3*.

### 2.8. Seahorse Metabolic Assays

A Seahorse Bioscience XF96 extracellular flux analyzer (Boston, MA, USA) was used to monitor oxygen consumption in accordance with the manufacturer’s instructions. The experiments were performed on day 6 of cell differentiation at passage 3. The oxygen consumption rate was assessed in preadipocytes that were overexpressed, silenced, or activated. After the administration of the complex III inhibitor antimycin A/rotenone, the mitochondrial uncoupled carbonyl cyanide-4-(trifluoromethoxy)-phenylhydrazone, or the ATP synthase inhibitor oligomycin respiration, was monitored under basal circumstances.

### 2.9. Western Blotting

Using RIPA lysis buffer (Solarbio, Beijing, China), total protein was extracted from the preadipocytes. Subsequently, the protein was separated on SDS-PAGE gels and transferred to PVDF membranes (Millipore, Billerica, MA, USA), and chemiluminescence detection for image capture was conducted using ChemStudio SA (Analytique, Jena, Germany). The antibodies used here mainly included: FABP4 (1:2000, ab92501, Abcam, Cambridge, MA, USA), PPARγ (1:1500, ab17886, Abcam, Cambridge, MA, USA), CEBPα (1:2000, ab40761, Abcam, Cambridge, MA, USA), PPARγ (1:1500, ab17886, Abcam, Cambridge, MA, USA), β-Tubulin (1:2000, ab179551, Abcam, Cambridge, MA, USA), PGC1α (1:1500, ab77210, Abcam, Cambridge, MA, USA), DIO2 (1:2000, ab77779, Abcam, Cambridge, MA, USA), and UCP3 (1:1500, ab61299, Abcam, Cambridge, MA, USA).

### 2.10. Fluorescence Microscopy

The cells were seeded, cultured, and differentiated into 6-well plates. The mitochondria were visualized by washing the live cells with warm media and incubating them with MitoTracker Red CMXRos (50 nM, Beyotime, Beijing, China) for 20 min, followed by fixation with 4% paraformaldehyde. Additionally, the lipid droplets were stained by incubating the fixed cells with BODIPY 493/503 (1:200) for 30 min at 18 °C after permeabilization with 0.1% Triton X-100 for 5 min. Subsequently, an antifade mounting medium with DAPI was added to the slides, and images were captured using a microscope (DP40; OLYMPUS, Tokyo, Japan).

### 2.11. mRNA Sequencing, RNA-seq Data Analysis, and Functional Analysis

Sequencing libraries and RNA-seq were conducted by Allwegene Biotechnology Co., Ltd. (Beijing, China). A cDNA library was constructed using RNA samples of high purity (OD 260/280 ≥ 2.0) and high integrity (RIN > 7). Differentially expressed genes (DEGs) were identified based on a fold change of (FC) > 1.5 and *p* < 0.05 after correcting for multiple testing. Gene ontology (GO) and Kyoto Encyclopedia of Genes and Genomes (KEGG) enrichment analyses were performed to elucidate the functions of the DEGs. The interactions between the DEGs were revealed using the Search Tool for the Retrieval of Interacting Genes/Proteins (STRING) with default parameters, and the results were visualized using Cytoscape. Furthermore, gene set enrichment analysis (GSEA) was utilized to evaluate the enriched pathways in each comparative group, and statistical analysis and mapping were conducted using an online website (https://cloud.oebiotech.cn/task/, accessed on 16 October 2023).

### 2.12. Statistical Analysis

All the experiments were conducted with a minimum of three biological replicates. Statistical analysis was performed using GraphPad Prism 6.01, and significance was calculated using the Student’s t test and one-way ANOVA (* *p* < 0.05, ** *p* < 0.01, *** *p* < 0.001, **** *p* < 0.0001). The data are presented as the mean ± standard error of the mean (SEM).

## 3. Results

### 3.1. Cold Stimulation Induces Browning of Subcutaneous Adipose Tissue in Min Pigs through Adrenoceptors

**Figure 1:**
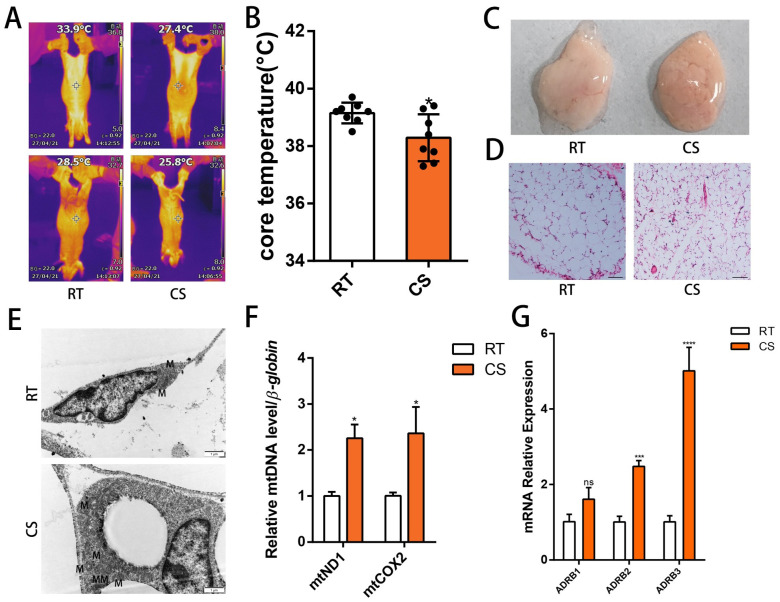
Cold stimulation of beige-colored subcutaneous white adipose tissue of *Min pigs* via adrenergic receptors. (**A**) Typical infrared thermograms of 30-day-old *Min pigs* under room temperature (RT, temperature = 25 °C) and cold stimulation (CS, temperature = 4 °C) treatments (*n* = 4–6). (**B**) Core temperature of 30-day-old *Min pigs* under RT and CS treatments (*n* = 4–6). (**C**,**D**) Representative images and H&E staining of subcutaneous adipose tissue from 30-day-old civilian pigs under RT (25 °C) and CS (4 °C) treatments. Scale bar: 100 mm. (**E**) Transmission electron microscopy images of inguinal adipose tissue from 30-day-old minipigs under RT and CS treatments, and M for mitochondria. Scale bar, 1 μm. (**F**) Relative mtDNA content of Min pigs under RT and CS treatments (*n* = 4–6). (**G**) Relative expression of *ADRB1*, *ADRB2*, and *ADRB3* in the RT and CS groups of Min pigs (*n* = 4–6). (* *p* < 0.05, *** *p* < 0.001, **** *p* < 0.0001).

To investigate the effects of cold stimulation on *Min pigs*, 30-day-old piglets were placed in metabolic cages for exposure to cold and room temperature conditions. Infrared photographs revealed a decrease in both body surface temperature and core temperature during cold stimulation. Interestingly, no significant temperature difference was observed between the axillary and inguinal regions, suggesting that these areas generated the most heat (Figure 1A,B). To explore whether the change in metabolic level caused by cold stimulation was due to the influence of non-shivering thermogenesis, we collected the inguinal subcutaneous adipose tissue of the *Min pigs* under cold stimulation and room temperature conditions. The color of the subcutaneous adipose tissue of the Min pigs significantly deepened under cold stimulation, and H & E staining revealed that the adipose tissue structure was denser, with a smaller diameter, and had increased small lipid droplets and multi-compartment adipocytes (Figure 1C,D). Subsequently, we found that the number of adipose tissue mitochondria increased and that the mitochondrial copy number increased under cold stimulation (Figure 1E,F). Because cold stress can activate the release of epinephrine, we further measured the expression levels of the adrenoceptors and found that *ADRB1*, *ADRB2*, and *ADRB3* showed different levels of increase (Figure 1G).

### 3.2. The Effect of Knocking down β-Adrenergic Receptors on Adipogenic Differentiation of Preadipocytes

**Figure 2:**
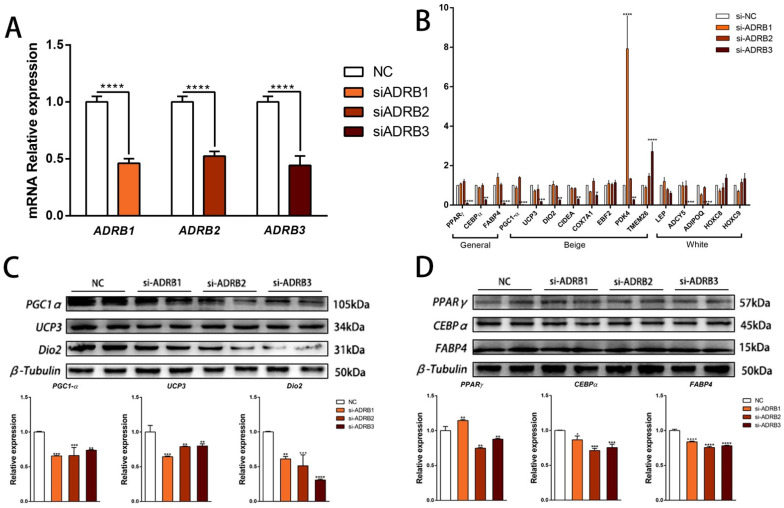
Effects of *ADRB1*, *ADRB2*, and *ADRB3* knockdown on the lipogenic differentiation of preadipocytes. (**A**) Interference efficiency of *ADRB1*, *ADRB2*, and *ADRB3* (*n* = 4–6). (**B**) Relative expression levels of adipocyte marker genes after the knockdown of *ADRB1*, *ADRB2*, and *ADRB3* (*n* = 4–6). (**C**) Western blot analysis of the levels of beige adipocyte marker genes *PGC1-α*, *UCP3*, and *Dio2* after the knockdown of *ADRB1*, *ADRB2*, and *ADRB3* (*n* = 4–6). (**D**) Western blot analysis of the levels of adipogenic marker genes *PPARγ*, *CEBPα*, and *FABP4* after the knockdown of *ADRB1*, *ADRB2*, and *ADRB3* (*n* = 4–6). (* *p* < 0.05, ** *p* < 0.01, *** *p* < 0.001, **** *p* < 0.0001).

**Figure 3:**
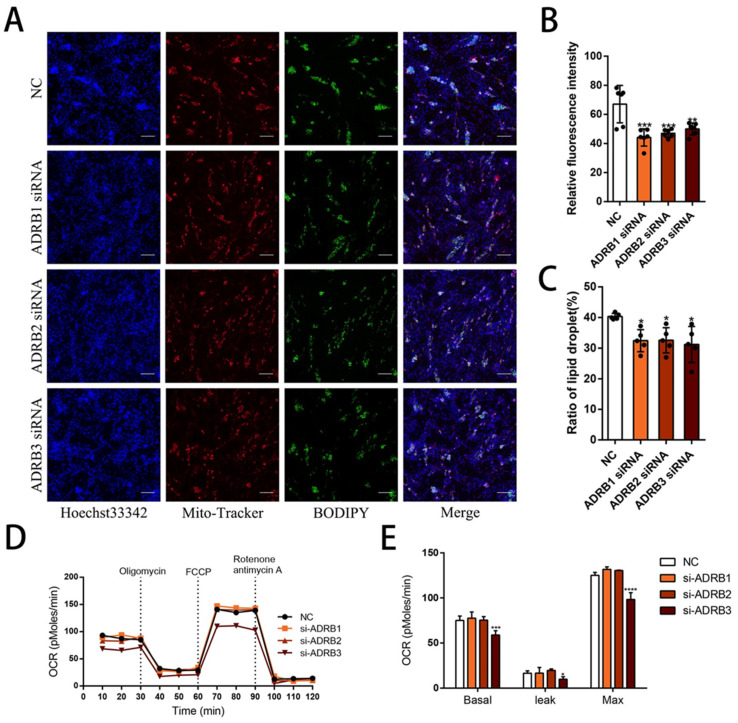
Effects of *ADRB1*, *ADRB2*, and *ADRB3* knockdown on energy production in preadipocytes. (**A**–**C**) MitoTracker detection of mitochondrial fluorescence and BODIPY staining for lipid formation levels (*n* = 4–6), Scale bar: 200 μm. (**D**,**E**) Measurement of oxygen consumption rates (OCR) of *ADRB1*, *ADRB2*, and *ADRB3* (*n* = 4–6). (* *p* < 0.05, ** *p* < 0.01, *** *p* < 0.001, **** *p* < 0.0001).

To determine the specific effects of each receptor on adipocyte differentiation, we conducted siRNA interference experiments on *ADRB1*, *ADRB2*, and *ADRB3* to assess their specific roles during preadipocyte differentiation (Figure 2A). We then evaluated the impact of the three receptors on preadipocyte beige differentiation by measuring the mRNA and protein expression levels using fluorescence quantitative PCR and WB, respectively. Knockdown of the *ADRB3* expression led to decreased mRNA and protein levels of the adipogenic marker genes. Additionally, the expression levels of six out of eight marker genes for beige adipocytes decreased at both the mRNA and the protein levels. However, knockdown of *ADRB1* and *ADRB2* did not show consistent effects on the mRNA and protein expression levels (Figure 2B–D). Following the evaluation of the mRNA and protein expression levels of the adipogenesis marker genes, we quantified the number of lipid droplets using fluorescence microscopy. The results showed that knocking down the three genes reduced both the rate of lipid formation and the fluorescence intensity of the mitochondria (Figure 3A–C). Furthermore, we used the Seahorse XF extracellular flux analyzer to assess the effect of knocking down the three receptor genes on cell metabolism. We observed a reduction in basic mitochondrial respiration and maximum mitochondrial respiratory capacity in the differentiated adipocytes after *ADRB3* knockdown, as well as a decrease in oligomycin-dependent uncoupled cell respiration (Figure 3D,E). These results indicate that *ADRB3* plays a role in enhancing preadipocyte metabolism.

### 3.3. The Effect of Overexpression of β-Adrenergic Receptors on Adipogenic Differentiation of Preadipocytes

**Figure 4:**
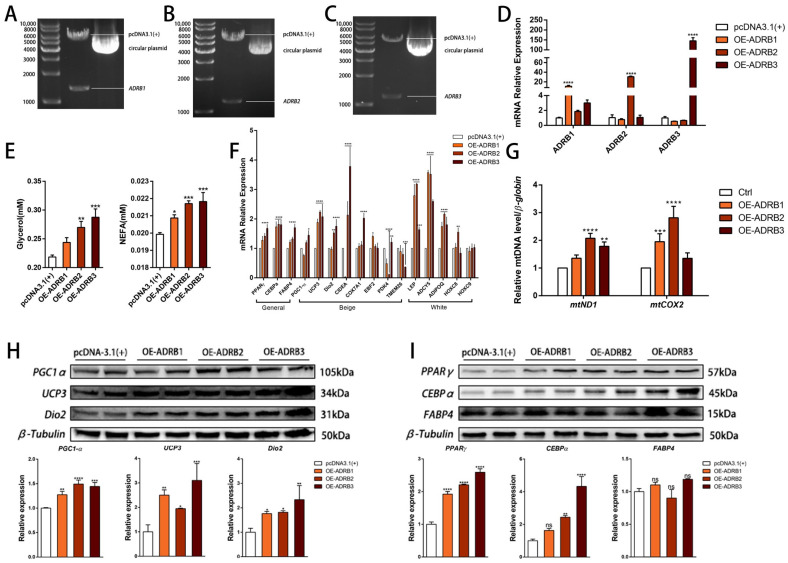
Effect of ADRB1, ADRB2, and ADRB3 overexpression on the lipogenic differentiation of preadipocytes. (**A**–**D**) Construction of pcDNA3.1(+)-*ADRB1*, pcDNA3.1(+)-*ADRB2*, and pcDNA3.1(+)-*ADRB3* overexpression vectors and detection of the relative expression of ADRB1, ADRB2, and ADRB3 by RT-qPCR (*n* = 4–6). (**E**) Free glycerol and NEFA levels in preadipocytes after the overexpression of *ADRB1*, *ADRB2*, and *ADRB3*. (**F**) Relative expression levels of adipocyte marker genes after overexpression of *ADRB1*, *ADRB2*, and *ADRB3* (*n* = 4–6). (**G**) Relative mtDNA content after overexpression of *ADRB1*, *ADRB2*, and *ADRB3* (*n* = 4–6). (**H**) Western blot analysis of the levels of beige adipocyte marker genes *PGC1-α*, *UCP3*, and *Dio2* after the overexpression of *ADRB1*, *ADRB2*, and *ADRB3* (*n* = 4–6). (**I**) Western blot analysis of the levels of adipogenic marker genes *PPARγ*, *CEBPα*, and *FABP4* after overexpression of *ADRB1*, *ADRB2*, and *ADRB3* (*n* = 4–6). (* *p* < 0.05, ** *p* < 0.01, *** *p* < 0.001, **** *p* < 0.0001).

**Figure 5:**
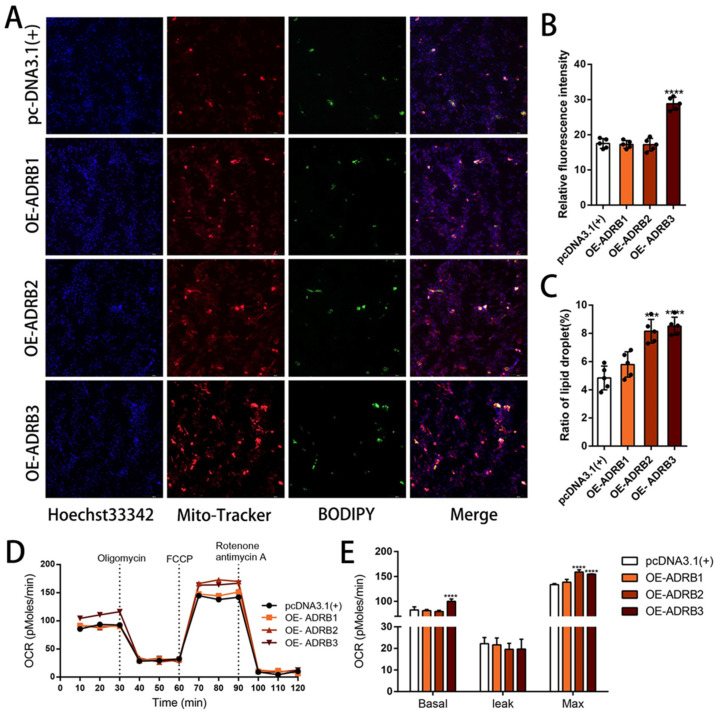
Effect of overexpression of *ADRB1*, *ADRB2*, and *ADRB3* on energy production in preadipocytes. (**A**–**C**) MitoTracker detection of mitochondrial fluorescence and BODIPY staining for lipid formation levels (*n* = 4–6), Scale bar: 200 μm. (**D**,**E**) Measurement of oxygen consumption rates (OCR) of *ADRB1*, *ADRB2*, and *ADRB3* overexpression (*n* = 4–6). (*** *p* < 0.001, **** *p* < 0.0001).

We constructed three overexpression vectors, pcDNA3.1 (+)—*ADRB1*, pcDNA3.1 (+)—*ADRB2*, and pcDNA3.1 (+)—*ADRB3* (Figure 4A–C), and transfected the overexpression plasmids into cells on the sixth day of differentiation. Subsequently, we conducted a validation analysis of the transfected cells, resulting in a 20–150-fold increase in the expression of the 3 genes (Figure 4D), confirming the successful construction and transfection of the vectors. To evaluate the lipolysis effect of the three genes on the cells, we detected the levels of free glycerol and NEFA and found that the overexpression of the three receptors promoted lipolysis (Figure 4E). Subsequently, we measured the beige cell levels after overexpression of the three genes. We observed an increase in the mRNA and protein levels of the adipogenic marker genes. Notably, the overexpression of *ADRB3* increased the expression of some beige adipocyte marker genes, including *PGC1-α*, *UCP3*, and *Dio2*. Interestingly, *ADRB3* also promoted the expression of four of the five white adipocyte marker genes (Figure 4F,H,I). Next, we evaluated the mitochondrial levels after overexpression of the three receptor genes. The fluorescence images of the preadipocytes showed that overexpression of *ADRB3* increased the fluorescence intensity of the mitochondria (Figure 5A,B), whereas *ADRB2* and *ADRB3* increased the levels of the mitochondrial copy numbers (Figure 4G). BODIPY staining showed that the level of the lipid droplets was similar to the gene expression level, with both *ADRB2* and *ADRB3* increasing the lipid droplets (Figure 5A,C). Additionally, overexpression of *ADRB3* enhanced mitochondrial respiration and OCR (Figure 5D,E), proving that *ADRB3* plays a unique role compared to the other two receptors.

### 3.4. ADRB3 Plays a Role in Preadipocyte Differentiation at the Transcriptome Level

**Figure 6:**
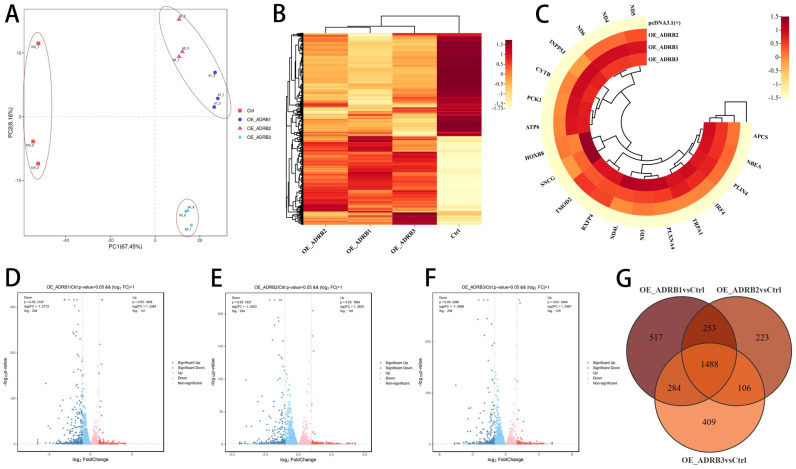
Identification of primary adrenergic receptors based on transcriptome sequencing. (**A**) Principal component analysis (PCA) plots based on transcriptome data. (**B**) Clustering heatmap between groups based on transcriptome data after overexpression of *ADRB1*, *ADRB2*, and *ADRB3*. (**C**) Heat map of the top 20 most differentially expressed genes after *ADRB3* overexpression. (**D**–**F**) Volcano plot showing a global overview of gene expression profiles in adipocytes after overexpression of *ADRB1*, *ADRB2*, and *ADRB3*. (**G**) The Venn plot shows overlapping genes with significant changes in each group after the overexpression of *ADRB1*, *ADRB2*, and *ADRB3*.

To determine the molecular mechanism of the adrenoceptors during preadipocyte differentiation, the preadipocytes cultured for 6 days were transfected with overexpression plasmids, and the cells were collected on day 7. Principal component analysis showed that *ADRB3* was clearly distinct from *ADRB1* and *ADRB2*, indicating different expression patterns (Figure 6A). The DEG clustering heat map of the four treatment groups also showed significant differences in gene expression patterns compared to the control group, with *ADRB1* and *ADRB2* classified together and *ADRB3* as a separate category (Figure 6B). We screened the top 20 major genes with differences, including 6 genes related to mitochondrial synthesis and genes related to lipid droplet synthesis (Figure 6C). Further analyses of the three treatment and control groups were conducted. By drawing a volcano map, the number of genes in each group was compared with that in the control group (Figure 6D–F). A Venn map was used to screen for unique and common gene change entries (Figure 6G). A follow-up analysis of the *ADRB3* regulated genes was performed to verify their unique role compared to the other two receptors.

### 3.5. Regulatory Network of ADRB3 on Preadipocyte Differentiation

**Figure 7:**
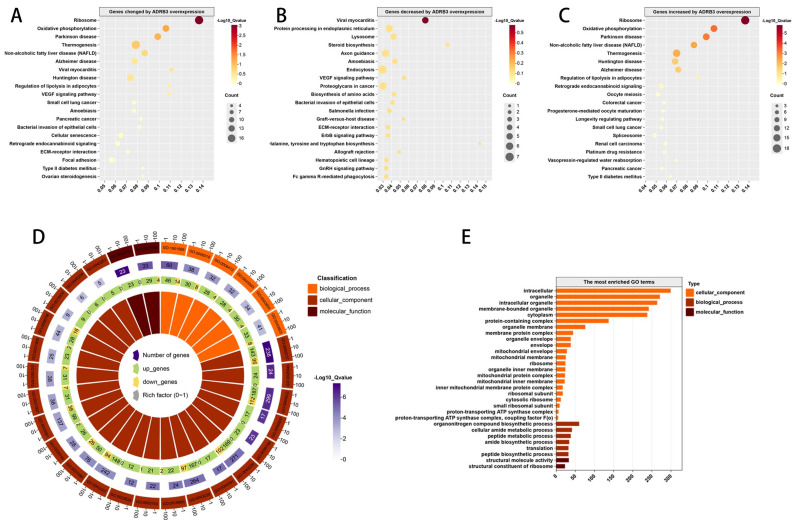
Gene regulatory network for the effect of *ADRB3* on preadipocyte differentiation. (**A**–**C**) KEGG pathway enrichment analysis of overexpressed ADRB3 differential genes; dot plots show the most significantly enriched pathways. The color of the dots represents the Q value, and the size of the dots represents the number of differentially expressed transcripts. (**D**,**E**) GO analysis of the overexpressed *ADRB3* differential genes.

**Figure 8:**
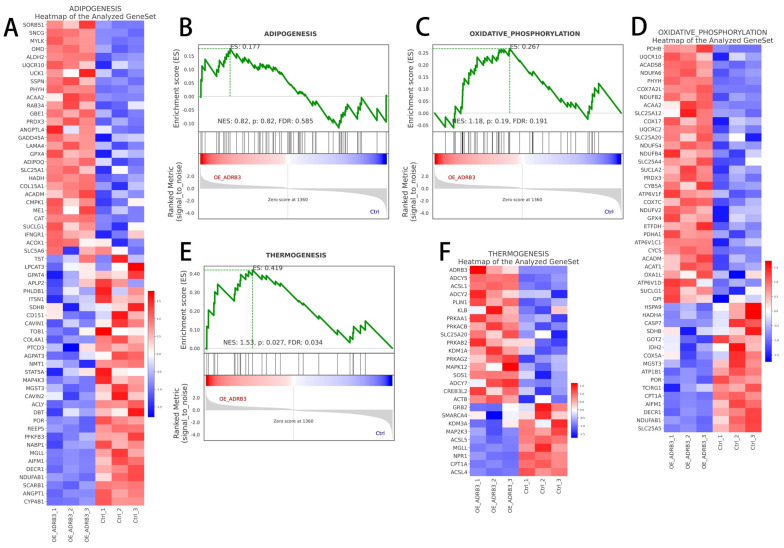
Gene regulatory network for the effect of *ADRB3* on preadipocyte differentiation. (**A**–**F**) Gene set enrichment analysis (GSEA) of RNA–seq data. Overexpression of *ADRB3* resulted in enrichment of the oxidative phosphorylation pathway, thermogenesis pathway, and adipogenesis pathway compared to controls.

To analyze the regulatory mode of *ADRB3* on preadipocyte differentiation further, we identified, through KEGG analysis, which pathways the *ADRB3*-changed genes, increased genes, and decreased genes were involved in, revealing their involvement in the oxidative phosphorylation pathway, thermogenic pathway, and adipogenesis pathway (Figure 7A–C). To determine the function of the DEGs, we used GO annotations to classify all DEG functions. The results showed the enrichment of 6 biological processes, with the most abundant being the organic compound biological process, 22 cellular components, with the most abundant being intracellular, and 2 molecular functions, with the most abundant being structural molecular activity (Figure 7D,E). In the GO enrichment analysis, we found that the expressions of most of the genes were upregulated in all the functions, indicating that the overexpression of *ADRB3* played a positive regulatory role in functional expression. Subsequently, GSEA was used to analyze the three pathways focused on the oxidative phosphorylation, thermogenic, and adipogenesis pathways (Figure 8A–F), indicating that *ADRB3* overexpression could upregulate the expression of these pathways.

## 4. Discussion

Cold stimulation excites the sympathetic nerve, causing the release of epinephrine and norepinephrine in the adrenal medulla [24]. These hormones then bind to different adrenoceptors, triggering a cascade of reactions. Our research confirmed that in Min pigs, the activation of *β3-AR* can resist cold by activating beige adipose tissue.

As a pig breed that thrives in cold regions, *Min pigs* exhibit better cold resistance characteristics compared to other breeds. These include a stronger metabolism under cold stress, minimal frostbite even at extremely cold temperatures, and the ability to maintain their core temperature [25,26,27]. Traditionally, pigs were thought to have poor cold resistance due to the loss of exons 3–5 of the *UCP1* gene and the absence of brown adipose tissue [28,29,30]. Therefore, Min pigs have significant research value in the field of cold resistance in pigs. In our study, we observed a decrease in body surface and core temperature in one-month-old *Min pigs* under cold stress, confirming their lack of BAT and cold tolerance and necessitating a constant-temperature heated production bed before weaning. Importantly, a decrease in body temperature does not equate to reduced metabolism. Indeed, the observation of adipose tissue confirmed this hypothesis, as cold stimulation turned the inguinal adipose tissue beige, which explains the highest temperature observed in the inguinal area in infrared images. Cold stimulation activates sympathetic nerve cells to release adrenaline, which acts on adrenaline receptors in beige adipose tissue, thereby activating the adrenergic signaling pathway. This process promotes the transcriptional regulation of *PGC1α*, *UCP3*, and *CIDEA* genes by *PPAR* and its co-regulatory factors. In summary, our study revealed that cold stimulation increased the expression of *β*-adrenoceptors and activated the thermogenic pathway, although the specific receptor responsible remains unknown.

We observed that *ADRB3* induced the browning of white adipocytes in a manner similar to that induced by cold stimulation, in contrast to the findings of Jiang et al., who suggested that low temperature and Adrb3 agonists activate distinct cellular populations [31]. This was evident not only in the increased expression levels of beige adipocyte marker genes at the mRNA and protein levels but also in the heightened mitochondrial levels, including an increase in mitochondrial copy numbers and enhanced mitochondrial fluorescence. Mitochondria play a crucial role in cellular respiratory metabolism by producing ATP through oxidative phosphorylation to supply energy to cells [32,33]. Our study demonstrated that *ADRB3* exhibited greater oxygen consumption and respiratory metabolic capacity, which in turn promoted lipolysis. *ADRB3* also increased the number of lipid droplets, causing a transition from large to small droplets. This change can be attributed to the browning of white adipocytes, which have a smaller lipid structure than white adipocytes, and lipolysis [34,35,36]. Our findings validate this perspective. However, the roles of *ADRB1* and *ADRB2* in the browning of white adipocytes were not consistent in our study, making it challenging to ascertain their specific contributions.

Due to its specific impact on the generation of beige adipocytes, *ADRB3*, as revealed by transcriptome sequencing analysis, was categorized separately from the other two receptors and the control group based on the principal component analysis (PCA) results. Notably, the cluster analysis results also indicated a closer classification relationship between the other two receptors, further validating the aforementioned findings. Notably, KEGG analysis demonstrated that *ADRB3* was enriched in the oxidative phosphorylation, thermogenic, and adipogenesis pathways compared to the control group. The oxidative phosphorylation pathway supplies energy and substrates for the adipogenesis pathway, whereas the adipogenesis pathway provides energy storage for the oxidative phosphorylation pathway [37]. Furthermore, the thermogenic pathway generates additional heat by activating the oxidative phosphorylation pathway and lipolysis in cold environments [38,39]. GSEA further confirmed our findings by indicating upregulation of the expression of the oxidative phosphorylation, thermogenic, and adipogenesis pathways.

Our experiments focused solely on the direct mechanism of action of *ADRB3* on beige fat production during cold exposure. However, it is important to note that there are many non-coding RNAs that also regulate beige fat production in cold exposure. One such example is miR-27 [40], which serves as an upstream central regulator of the transcriptional network involved in beige and brown lipogenesis following cold exposure. Further experimental evidence is needed to determine whether *ADRB3* affects beige fat production by regulating miR-27.

When the body is subjected to cold stress, the sympathetic nervous system releases adrenaline, which acts on adrenergic receptors to regulate various physiological functions, such as the browning of white adipose cells. In experiments simulating cold stimulation in preadipocytes, norepinephrine (NE) as a ligand can mimic the effects of cold stimulation [41]. However, compounds like Formoterol, an *ADRB2* agonist, and Mirabegron, an *ADRB3* agonist, also serve to simulate cold stress experiments, despite not being neurotransmitters [42,43]. Nevertheless, they can increase the quantity of *ADRB2* and *ADRB3*. Therefore, we infer that cold stimulation leads to an increase in both the neurotransmitter adrenaline and the quantity of adrenergic receptors, with the latter playing a decisive role. Nonetheless, to fully demonstrate the regulation of the browning of adipose tissue by *ADRB3*, the optimal approach would still be to conduct gene knockout or overexpression experiments in vivo; to strengthen our conclusions, this will be the focus of our further research.

In conclusion, our study shows that cold stimulation can cause the beige discoloration of inguinal WAT in Min piglets via adrenoceptors. *ADRB3* inhibits the proliferation of preadipocytes and plays a key role in promoting lipolysis, heat production, and beige cells during adipogenesis. Furthermore, transcriptome analysis revealed that *ADRB3*, rather than *ADRB1* or *ADRB2*, played a key regulatory role in thermogenesis and adipogenesis. Our research identified a crucial gene for cold resistance in Min pigs, providing theoretical support for cold resistance breeding in pigs. Additionally, we discovered a reliable model for combating obesity and related metabolic diseases in humans.

## Data Availability

The data that support this study are provided in the main text or the Supplementary Materials. All raw sequencing data were deposited to the NCBI resource under the accession number PRJNA1096254. All the relevant data are provided along with the manuscript as Supplementary Files.

## Supplementary Materials

The following supporting information can be downloaded at: https://www.mdpi.com/article/10.3390/cells13080709/s1, Table S1: The primers for qPCR, Table S2: pcDNA3.1 (+)—ADRB1, pcDNA3.1 (+)—ADRB2, and pcDNA3.1 (+)—ADRB3 primer sequences, Table S3: siRNA sequences.

## References

[1] Cannon B., Nedergaard J. (2004). Brown adipose tissue: Function and physiological significance. Physiol. Rev..

[2] Spiegelman B.M., Flier J.S. (2001). Obesity and the regulation of energy balance. Cell.

[3] Hafner A.-L., Dani C. (2014). Human induced pluripotent stem cells: A new source for brown and white adipocytes. World J. Stem Cells.

[4] Yao X., Dani V., Dani C. (2020). Human Pluripotent Stem Cells: A Relevant Model to Identify Pathways Governing Thermogenic Adipocyte Generation. Front. Endocrinol..

[5] Pearson S., Loft A., Rajbhandari P., Simcox J., Lee S., Tontonoz P., Mandrup S., Villanueva C.J. (2019). Loss of *TLE3* promotes the mitochondrial program in beige adipocytes and improves glucose metabolism. Genes. Dev..

[6] Kajimura S., Spiegelman B.M., Seale P. (2015). Brown and Beige Fat: Physiological Roles beyond Heat Generation. Cell Metab..

[7] Harms M., Seale P. (2013). Brown and beige fat: Development, function and therapeutic potential. Nat. Med..

[8] Wang Q.A., Tao C., Gupta R.K., Scherer P.E. (2013). Tracking adipogenesis during white adipose tissue development, expansion and regeneration. Nat. Med..

[9] Barbatelli G., Murano I., Madsen L., Hao Q., Jimenez M., Kristiansen K., Giacobino J.P., De Matteis R., Cinti S. (2010). The emergence of cold-induced brown adipocytes in mouse white fat depots is determined predominantly by white to brown adipocyte transdifferentiation. Am. J. Physiol.-Endocrinol. Metab..

[10] Himms-Hagen J., Melnyk A., Zingaretti M.C., Ceresi E., Barbatelli G., Cinti S. (2000). Multilocular fat cells in WAT of CL-316243-treated rats derive directly from white adipocytes. Am. J. Physiol.-Cell Physiol..

[11] Zhang D., Ma S., Wang L., Ma H., Wang W., Xia J., Liu D. (2022). Min pig skeletal muscle response to cold stress. PLoS ONE.

[12] Liu Y.Z., Yang X.Q., Jing X.Y., He X.M., Wang L., Liu Y., Liu D. (2018). Transcriptomics Analysis on Excellent Meat Quality Traits of Skeletal Muscles of the Chinese Indigenous Min Pig Compared with the Large White Breed. Int. J. Mol. Sci..

[13] Hao W.J., Yang Z.W., Sun Y.L., Li J.X., Zhang D.J., Liu D., Yang X.Q. (2022). Characterization of Alternative Splicing Events in Porcine Skeletal Muscles with Different Intramuscular Fat Contents. Biomolecules.

[14] Lin J., Cao C.W., Tao C., Ye R.C., Dong M., Zheng Q.T., Wang C., Jiang X.X., Qin G.S., Yan C.G. (2017). Cold adaptation in pigs depends on *UCP3* in beige adipocytes. J. Mol. Cell Biol..

[15] Zhang L.L., Hu S.L., Cao C.W., Chen C.H., Liu J.L., Wang Y., Liu J.F., Zhao J.G., Tao C., Wang Y.F. (2022). Functional and Genetic Characterization of Porcine Beige Adipocytes. Cells.

[16] Lu M., He Y., Gong M., Li Q., Tang Q., Wang X., Wang Y., Yuan M., Yu Z., Xu B. (2020). Role of Neuro-Immune Cross-Talk in the Anti-obesity Effect of Electro-Acupuncture. Front. Neurosci..

[17] Srivastava R.K., Ruiz de Azua I., Conrad A., Purrio M., Lutz B. (2022). Cannabinoid *CB1* Receptor Deletion from Catecholaminergic Neurons Protects from Diet-Induced Obesity. Int. J. Mol. Sci..

[18] Kouidhi M., Villageois P., Mounier C.M., Ménigot C., Rival Y., Piwnica D., Aubert J., Chignon-Sicard B., Dani C. (2015). Characterization of human knee and chin adipose-derived stromal cells. Stem Cells Int..

[19] Kurylowicz A., Jonas M., Lisik W., Jonas M., Wicik Z.A., Wierzbicki Z., Chmura A., Puzianowska-Kuznicka M. (2015). Obesity is associated with a decrease in expression but not with the hypermethylation of thermogenesis-related genes in adipose tissues. J. Transl. Med..

[20] Collins S., Surwit R.S. (2001). The beta-adrenergic receptors and the control of adipose tissue metabolism and thermogenesis. Recent Prog. Horm. Res..

[21] Shin S., Ajuwon K.M. (2018). Divergent Response of Murine and Porcine Adipocytes to Stimulation of Browning Genes by 18-Carbon Polyunsaturated Fatty Acids and Beta-Receptor Agonists. Lipids.

[22] Shin S., Ajuwon K.M. (2017). Activation of Adrenergic Receptor Subtypes Differentially Regulate Expression of Metabolic Genes in Porcine Adipocytes. FASEB J..

[23] Mills S. (2000). Beta-adrenergic receptor subtypes mediating lipolysis in porcine adipocytes. Studies with BRL-37344, a putative beta3-adrenergic agonist. Comp. Biochem. Physiol. Toxicol. Pharmacol. CBP.

[24] Berretz G., Packheiser J., Wolf O.T., Ocklenburg S. (2022). Acute stress increases left hemispheric activity measured via changes in frontal alpha asymmetries. iScience.

[25] Sun Y.L., Lin X., Zhang Q., Pang Y., Zhang X.H., Zhao X.L., Liu D., Yang X.Q. (2022). Genome-wide characterization of lncRNAs and mRNAs in muscles with differential intramuscular fat contents. Front. Vet. Sci..

[26] Yang Y., Sun C.C., Li F., Shan A.S., Shi B.M. (2021). Characteristics of faecal bacterial flora and volatile fatty acids in Min pig, *Landrace pig*, and *Yorkshire pig*. Electron. J. Biotechnol..

[27] Teng T., Sun G.D., Ding H.W., Song X., Bai G.D., Shi B.M., Shang T.T. (2023). Characteristics of glucose and lipid metabolism and the interaction between gut microbiota and colonic mucosal immunity in pigs during cold exposure. J. Anim. Sci. Biotechnol..

[28] Gaudry M.J., Jastroch M., Treberg J.R., Hofreiter M., Paijmans J.L.A., Starrett J., Wales N., Signore A.V., Springer M.S., Campbell K.L. (2017). Inactivation of thermogenic UCP1 as a historical contingency in multiple placental mammal clades. Sci. Adv..

[29] Berg F., Gustafson U., Andersson L. (2006). The uncoupling protein 1 gene (*UCP1*) is disrupted in the pig lineage: A genetic explanation for poor thermoregulation in piglets. PLoS Genet..

[30] Zhou Y., Xu Z., Wang L., Ling D., Nong Q., Xie J., Zhu X., Shan T. (2022). Cold Exposure Induces Depot-Specific Alterations in Fatty Acid Composition and Transcriptional Profile in Adipose Tissues of Pigs. Front. Endocrinol..

[31] Jiang Y.W., Berryit D.C., Grain J.M. (2017). Distinct cellular and molecular mechanisms for *β3* adrenergic receptor-induced beige adipocyte formation. eLife.

[32] Heazlewood J.L., Tonti-Filippini J.S., Gout A.M., Day D.A., Whelan J., Millar A.H. (2004). Experimental analysis of the Arabidopsis mitochondrial proteome highlights signaling and regulatory components, provides assessment of targeting prediction programs, and indicates plant-specific mitochondrial proteins. Plant Cell.

[33] Moreno-Sánchez R., Marín-Hernández A., Saavedra E., Pardo J.P., Ralph S.J., Rodríguez-Enríquez S. (2014). Who controls the ATP supply in cancer cells? Biochemistry lessons to understand cancer energy metabolism. Int. J. Biochem. Cell Biol..

[34] Colson C., Batrow P.L., Gautier N., Rochet N., Ailhaud G., Peiretti F., Amri E.Z. (2020). The Rosmarinus Bioactive Compound Carnosic Acid Is a Novel PPAR Antagonist That Inhibits the Browning of White Adipocytes. Cells.

[35] Woodall B.P., Gresham K.S., Woodall M.A., Valenti M.C., Cannavo A., Pfleger J., Chuprun J.K., Drosatos K., Koch W.J. (2019). Alteration of myocardial *GRK2* produces a global metabolic phenotype. JCI Insight.

[36] Kahoul Y., Yao X., Oger F., Moreno M., Amanzougarene S., Derhourhi M., Durand E., Boutry R., Bonnefond A., Froguel P. (2023). Knocking Down *CDKN2A* in 3D hiPSC-Derived Brown Adipose Progenitors Potentiates Differentiation, Oxidative Metabolism and Browning Process. Cells.

[37] Wang Z., Zhu S., Li C., Lyu L., Yu J., Wang D., Xu Z., Ni J., Gao B., Lu J. (2022). Gene essentiality profiling reveals a novel determinant of stresses preventing protein aggregation in Salmonella. Emerg. Microbes Infect..

[38] Fenzl A., Kiefer F.W. (2014). Brown adipose tissue and thermogenesis. Horm. Mol. Biol. Clin. Investig..

[39] Ladoux A., Peraldi P., Chignon-Sicard B., Dani C. (2021). Distinct Shades of Adipocytes Control the Metabolic Roles of Adipose Tissues: From Their Origins to Their Relevance for Medical Applications. Biomedicines.

[40] Sun L., Trajkovski M. (2014). MiR-27 orchestrates the transcriptional regulation of brown adipogenesis. Metab.-Clin. Exp..

[41] Blondin D.P., Nielsen S., Kuipers E.N., Severinsen M.C., Jensen V.H., Miard S., Jespersen N.Z., Kooijman S., Boon M.R., Fortin M. (2020). Human Brown Adipocyte Thermogenesis Is Driven by *β2-AR* Stimulation. Cell Metab..

[42] Cero C., Lea H.J., Zhu K.Y., Shamsi F., Tseng Y.H., Cypess A.M. (2021). β3-Adrenergic receptors regulate human brown/beige adipocyte lipolysis and thermogenesis. JCI Insight.

[43] Riis-Vestergaard M.J., Richelsen B., Bruun J.M., Li W., Hansen J.B., Pedersen S.B. (2020). Beta-1 and Not Beta-3 Adrenergic Receptors May Be the Primary Regulator of Human Brown Adipocyte Metabolism. J. Clin. Endocrinol. Metab..

